# Current and future advances in fluorescence-based visualization of plant cell wall components and cell wall biosynthetic machineries

**DOI:** 10.1186/s13068-021-01922-0

**Published:** 2021-03-29

**Authors:** Brian T DeVree, Lisa M Steiner, Sylwia Głazowska, Felix Ruhnow, Klaus Herburger, Staffan Persson, Jozef Mravec

**Affiliations:** 1 Department of Plant and Environmental Sciences, University of Copenhagen, Thorvaldsensvej 40, 1871 Frederiksberg, Denmark; 2 Joint International Research Laboratory of Metabolic and Developmental Sciences, State Key Laboratory of Hybrid Rice, School of Life Sciences and Biotechnology, Shanghai Jiao Tong University, Shanghai, China

**Keywords:** Plant cell wall architecture, Fluorescence microscopy, Superresolution microscopy, Live cell imaging, Cell wall probes, Nanobodies, Lambodies, Click chemistry, Genetic probes, Aptamers, Carbohydrate-binding modules, Anti-glycan antibodies

## Abstract

Plant cell wall-derived biomass serves as a renewable source of energy and materials with increasing importance. The cell walls are biomacromolecular assemblies defined by a fine arrangement of different classes of polysaccharides, proteoglycans, and aromatic polymers and are one of the most complex structures in Nature. One of the most challenging tasks of cell biology and biomass biotechnology research is to image the structure and organization of this complex matrix, as well as to visualize the compartmentalized, multiplayer biosynthetic machineries that build the elaborate cell wall architecture. Better knowledge of the plant cells, cell walls, and whole tissue is essential for bioengineering efforts and for designing efficient strategies of industrial deconstruction of the cell wall-derived biomass and its saccharification. Cell wall-directed molecular probes and analysis by light microscopy, which is capable of imaging with a high level of specificity, little sample processing, and often in real time, are important tools to understand cell wall assemblies. This review provides a comprehensive overview about the possibilities for fluorescence label-based imaging techniques and a variety of probing methods, discussing both well-established and emerging tools. Examples of applications of these tools are provided. We also list and discuss the advantages and limitations of the methods. Specifically, we elaborate on what are the most important considerations when applying a particular technique for plants, the potential for future development, and how the plant cell wall field might be inspired by advances in the biomedical and general cell biology fields.

## Background

Plant growth is based on the ability of plants to convert carbon dioxide into sugars via photosynthesis and metabolize them into a wide range of other biomolecules [1]. The main carbon sink in plants is the cell wall; an extracellular matrix composed of long-chain glycans, glycoproteins, phenolic and polyester polymers, as well as solutes and water. The cell walls provide important structural and protective functions to plants as well as contribute the bulk of their biomass [2]. A large number of products, such as biobased fuels, chemicals, paper, and novel materials may be derived from this biomass, and finding sustainable and carbon-neutral approaches to do this will be an important part of shifting our society away from a fossil-fuel based economy [3]. We anticipate that these efforts will be aided by a better understanding of how this biomass is structured, how it is created by the plant, and what happens as it is being processed, and discuss the toolset available to accomplish these studies using fluorescence microscopy in this review.

The major constituents of plant cell walls are polysaccharides, which are divided into three different classes: cellulose, hemicelluloses, and pectins [4–6]. Cellulose consists of $\beta$-1,4-linked glucose that coalesces into microfibrils via intermolecular hydrogen bonds and van der Waal’s forces. The cellulose microfibrils have a high tensile strength and work as a scaffold, providing the load-bearing strength to the cell walls [7, 8]. Cellulose is produced at the cell surface by cellulose synthase (CesA) protein complexes (CSCs), which utilize cytosolic UDP-glucose as their substrate [9, 10].

Hemicelluloses primarily consist of $\beta$-1,4-linked neutral sugar backbones with equatorial conformations and include xyloglucan, xylan, mannan, glucomannan, and mixed-linkage glucan [5]. These polymers are made in the Golgi lumen, with the possible exception of mixed-linked glucan [11–13], by glycosyltransferases (GTs) that use an array of nucleotide sugars as substrates. Hemicelluloses engage with cellulose and/or lignin to regulate, depending on the developmental context, either cell wall expansion and cell growth or cell wall rigidification [8, 14, 15].

Pectins are also made in the Golgi lumen by GTs and are some of the most complex and dynamic cell wall molecules. Homogalacturonan (HG), a homopolymer of $\alpha$-1,4-linked galacturonic acid, is synthesized in a highly methylesterified form and upon secretion in the apoplastic moiety can be de-esterified by a class of enzymes called pectin methylesterases (PMEs). The modulation of PME activity underlies cell wall-directed cellular and developmental processes, for instance, meristem formation or pavement cell morphogenesis [16–18]. HG backbone can be decorated with monosaccharides such as apiose (apiogalacturonan), xylose (xylogalacturonan), or by a complex assortment of sugars and glycosidic linkages known as rhamnoglacturonan II (RG-II). Another pectin with a backbone of repeating disaccharide of galacturonic acid and rhamnose units is rhamnogalacturonan I (RG-I), which is further modified with galactan and arabinan side chains. Methylation and acetylation of pectins provide further important molecular features that influence biomass processing and fermentability [19, 20].

Unlike the flexible primary cell walls which encase cells that are still growing, thick secondary cell walls are deposited once cells have ceased growth. These strong walls provide mechanical strength as well as creating the vascular tissue needed for water transport and providing resistance to biotic threats [21]. The secondary cell walls make up the bulk of a plant’s biomass and are the major source of fermentable sugars for cellulosic biofuel production [22]. A prominent component of many secondary cell walls is lignin, which is a highly heterogenous phenolic polymer that is polymerized directly in the cell wall by laccases and peroxidase-assisted radical coupling of small aromatic alcohols known as monolignols [23]. This extensive crosslinking reinforces the cell walls, but lignin itself also acts as an essential hydrophobic barrier on xylem vessels to enable long distance water transport.

Because of its abundance and extensive crosslinking, lignin is usually the main factor that influences the resistance of cell walls to decomposition. However, the recalcitrance of biomass to processing is still a poorly understood phenomenon that is also influenced by cell wall morphology, porosity, and the abundance of the varying constituent polymers [24–27]. Fluorescence-based imaging can be used to assess these features in both native cell walls and processed samples, showing things like cell wall microdomains, the accessibility of enzymatic machineries, or the effects of physical or chemical treatments on the sample [28, 29].

There are a handful of imaging methods that utilize the intrinsic chemical or mechanical features of cell wall polymers, such as atomic force microscopy (AFM), Fourier-transformed infrared (FTIR) microspectroscopy, confocal Raman microspectroscopy (CRM), coherent Anti-Stokes Raman scattering microscopy (CARS), stimulated Raman scattering microscopy (SRS), Brillouin microscopy, and X-ray computed tomography (CT) [29, 30]. Although these techniques are outside of the scope of this review, they are expected to be particularly useful for characterization of samples with unusual functional groups or electronic densities. Additionally, some fluorescence-based methods like Förster resonance energy transfer (FRET) microscopy and fluorescence lifetime imaging microscopy (FLIM) have recently been well summarized in other reviews [30, 31], so we do not discuss the special biophysical information they can provide here but note that they share many of the same challenges as standard fluorescent imaging and molecular tagging.

This review is divided into two major sections: the first part discusses options for cell wall visualization by various types of fluorescence microscopy and the second section deals with molecules that enable exogenous or endogenous ‘tagging’ specific molecular targets. All research methods come with their own limitations, drawbacks, and challenges, and we use this opportunity to particularly highlight these. We also suggest how future advances and development in the field can mitigate the current drawbacks and pitfalls.

## Fluorescence-based optical microscopy

### Basic epifluorescence and confocal microscopy

Epifluorescence microscopy and confocal laser-scanning microscopy (CLSM) are well-established microscopy techniques. Epifluorescence involves simultaneous illumination and detection of the entire field of view, allowing low photon doses and quick imaging for effective control of phototoxicity, but large amount of out-of-focus light is collected. CLSM has the opposite limitations, using a bright, focused excitation laser that is scanned across the sample, removing unwanted emission signal by passage of the returned light through a pinhole before detection. This gives crisp images, at the expense of high photon doses and slow imaging times. Basic CLSM or epifluorescence images can be particularly helpful when using superresolution imaging, as comparison of images made with these novel methods and well-understood, traditional techniques allows one to clearly determine what features should be evaluated as potential artifacts or as new discoveries. Spinning-disk confocal microscopy overcomes the disadvantage of traditional CLSM’s long image acquisition times and high illumination intensities while still rejecting a very large fraction of out-of-focus light by using multiple pinholes to project a series of moving, parallel excitation light beams onto the sample. These features make it ideal to study the dynamics of fast biological processes with much lower levels of photobleaching and phototoxicity.

### Deconvolution and widefield microscopy

Deconvolution techniques serve similar purposes to light restriction by confocal pinholes, reducing the amount of out-of-focus light in the resulting image. However, rather than blocking light, deconvolution reassigns it back to its true source by correcting for the dispersion of the signal via a (relatively difficult) data analysis optimization problem [32]. While it is possible to apply the technique to 2D-only images, a process often termed deblurring, it is most effective when used with a 3D stack of images taken at multiple focal planes [33]. Using deconvolution techniques along with widefield imaging is among the very best ways to avoid photodamage of live samples.

### Deconvolution and confocal microscopy

Confocal images do not have perfect out-of-focus light restriction, and datasets can be improved somewhat with appropriate deconvolution methods [34]. Additionally, several imaging techniques and microscopes now combine the principles of confocal microscopy and deconvolution to provide modest superresoution capabilities. One such class of techniques is known as image-scanning microscopy (ISM) [35], employed by instruments like the Zeiss Airyscan, which replaces the confocal detection pinhole with an array of photomultiplier tubes. A $\sqrt{2}$ resolution enhancement is realized by removing the tradeoff between lateral resolution and signal throughput inherent in pinhole-based instruments, and because the detector array physically collects an estimate of the Airy pattern of emitted light, deconvolution can be applied to further increase the superresolution capability up to a factor-of-2 limit [36]. On a spinning-disk confocal instrument, shaping the light passing through the pinholes with a microlens array and magnification of emitted light on the camera sensor by optical reassignment allows a similar factor-of-2 resolution enhancement when coupled with image deconvolution [37]. These types of enhanced confocal microscopes are increasingly used in plant cell research, to, for example, study plasma membrane nanodomains on live cells [38], or pollen morphology [39].

### Total internal reflection fluorescence microscopy

**Fig. 1:**
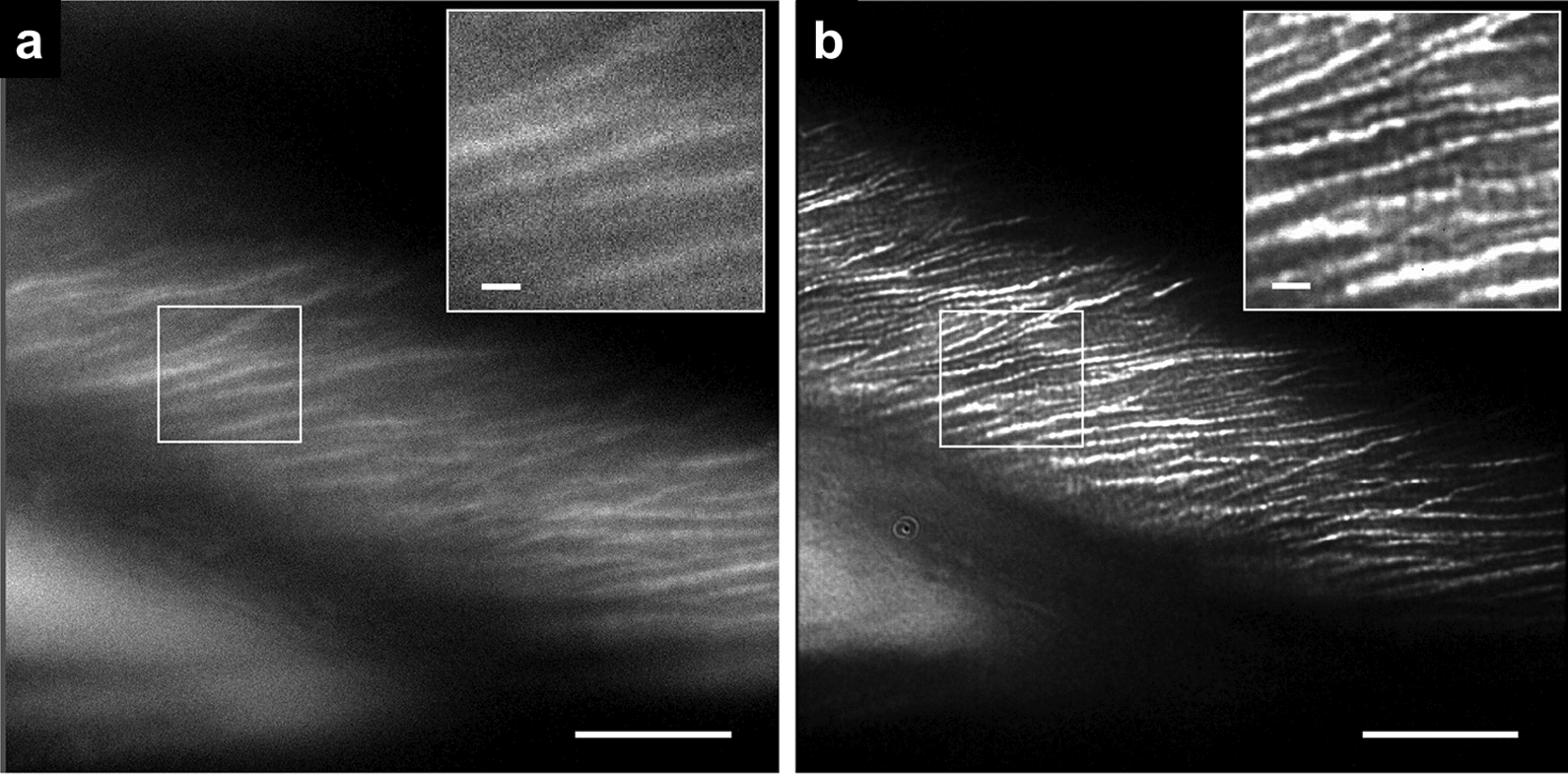
Superresolution images of Pontamine Fast Scarlet 4B-stained cellulose fibrils in onion bulb scale epidermis cells. **a** Total internal reflection fluorescence (TIRF) image of the cell surface **b** The section was then bleached using a strong laser light to achieve the recording of the single ’blinking’ events of the fluorophores. The image shows rendering of stochastic optical reconstruction microscopy (STORM) results. Note the much finer resolution of the cellulose fibrils. Scale bars 10, 1 μm in inserts (Reprint from [42]), CC BY 2.0

Total internal reflection fluorescence microscopy (TIRFM) provides the excitation energy as a thin ($\approx$100 nm) evanescent wavefront at the contact area between the sample and the cover slip, which allows outstanding image quality in this region. Additionally, variable-angle epifluorescence microscopy (VAEM) can be used on plant cell and cell wall samples thicker than the TIRF wavefront, with a similar reduction of background illumination [40]. Figure 1a shows an example of a TIRF scan of an onion epidermal cell with a cellulose-specific stain, showing a narrow optical section through the cell wall surface as well as supporting high-intensity illumination for superresolution imaging, shown in Fig. 1b. TIRFM is also fully compatible with live cell imaging, for example, it was used to study the CesA complexes’ movement along microtubules [41].

### Light sheet fluorescent microscopy

Light sheet fluorescent microscopy (LSFM) can be applied to image developmental processes in plant cells that are hidden under thick layers of plant tissues and that are difficult to physically extract (e.g., male and female germlines [43]). LSFM uses specialized two-objective optics that enables creation of a highly localized plane of illumination. This technique minimizes photodamage, has a high imaging speed which is comparable to spinning-disk microscopy ($\approx$100 fps), and allows 360° rotation of samples. Most importantly, it is compatible with live imaging over days [44] and can be used for plant samples with low expression of fluorescently tagged proteins. LSFM was recently used to image cellular processes within *Arabidopsis* flowers [45], and this technique is promising for studying synthesis and remodeling enzymes in the cell wall over long time periods. One of its drawbacks is that the typical required geometry of the two objective lenses set at 90° means that the x, y, and z resolution are restricted due to larger working distance requirements (e.g., z resolution in LSFM is $\approx$2 $\mu$m to $\approx$700 nm) [46].

### Superresolution microscopy overview

Even the best objective lenses can only laterally resolve light from visible wavelengths to around 200 nm, but individual proteins and glycans are typically one or two orders of magnitude smaller, 2 to 20 nm in width. In order to come closer to resolving the fine structure of cell walls, fluorescent imaging with superresolution techniques that overcome the resolution limit of a microscope’s optical system is becoming widely used in plant cell research (reviewed in [47, 48]). Some examples include the organization of cellulose microfibrils [42], or the role of putative pectin rods involved in lobe formation of leaf epidermal cells [16, 17].

It is essential for the user to understand that many superresolution images are a product of extensive data analysis and fitting and can be fundamentally different to those created with traditional optics. This issue is most pronounced for images produced with localization microscopy techniques, and less so for structured illumination microscopy (SIM) or stimulated emission depletion (STED) microscopy. This is quickly appreciated by considering what a dark area of an image is comprised of for normal or for localization-based images. With widefield, confocal, SIM, or STED fluorescence microscopy, a dark area on an image is physically measured to have no fluorescent signal at the corresponding location in the sample. However, for localization microscopy (as discussed below in "Localization-based superresolution methods" section), a dark spot may either have no fluorescent label, an irreversibly bleached or missing label, or a functional label molecule that simply has not happened to be activated out of its dark state yet. Similarly, it is easy for noise to inject location-fitting errors into the light regions of a localization microscopy image as well, leading to a situation where dark and light regions are only statistical estimates of what the true sample looks like. Elimination of or compensation for these effects is challenging, and unfortunately, they can cause the resulting images to contain artifacts [49]. The reader is directed to Baumgart et al. [50] for an entertaining presentation of how these artifacts can be created by the special process of localization microscopy.

### Structured illumination microscopy

In structured illumination microscopy (SIM) [51], the illumination pattern of a widefield fluorescent microscope is modified so that it arrives with a sine-wave interference pattern, which is then moved along the sample at several different phases. Data are collected with the illumination lines set at three or more different angles, and the images are combined into a single image with a maximum of twice the resolution that the microscope could normally obtain; this principle can be extended to 3D datasets or TIRF microscopy with optics designed to provide more complex illumination patterns. The resolution enhancements come from obtaining similar information as that recorded by the confocal-based image-scanning microscopy discussed above, but with a more efficient extraction of high-frequency spatial information [52]. SIM has been used to study association of microtubules and proteins [53], or cellulose microfibril orientation in onion cells [42].

### Stimulated emission depletion

Stimulated emission depletion (STED) microscopy is a superresolution technique based on confocal microscopy, but it uses a sophisticated two-laser shaped beam to effectively shrink the spot size of the scanning excitation laser [54]. While fairly simple in concept and suitable for many samples that can be imaged by traditional confocal microscopy, the technique requires quite advanced, expensive equipment and is frustrated in many plant cell samples which contain red autofluorescence and/or strong refractive index changes [47]. Despite these challenges, a recent STED study on plant cell walls with a PEG-rhodamine conjugate as a fluorescent probe for lignin demonstrated improved resolution for imaging of the middle lamella organization in wood [55].

### Expansion microscopy

Expansion microscopy (ExM) relies on physical expansion of biological structures by flooding a sample with acrylamide and then polymerizing the monomers into a hydrophilic polyacrylamide gel cross-linked to cellular components or fluorescent labels. The sample is then enzymatically digested and the polymer is carefully swollen from 4 to 10 times the original size, effectively allowing a 4-10x higher resolution image to be taken on completely standard imaging equipment. Application of this method to organisms with strong cell walls lags significantly behind its use with softer cell types, but there are a few reported examples. Several fungal species were recently successfully expanded [56], and the technique was also used in plants to image the chromatin ultrastructure in barley [57], and aspects of transcription regulation during *Arabidopsis* embryo fertilization [58]. With continued method development, this technique has a strong potential to enable nanoscale fluorescent imaging of otherwise inaccessible cell wall epitopes.

### Localization-based superresolution methods

A large number of fluorescent superresolution imaging techniques that have been developed in the last $\approx$15 years are based on estimation of a point-source fluorescent emitter’s position. Techniques like stochastic optical reconstruction microscopy (STORM), photo-activated localization microscopy (PALM), and many others share a large number of basic principles [59, 60]. Fundamentally, they all find a way to reduce the number of fluorophores detected in an image to a small fraction of the total, and then, the individual fluorophores are analyzed as single molecules with a defined spatial position. Many images are taken with different subsets of fluorophores activated, and then, the corresponding positions of the detected molecules are added together into a composite superresolution image. The process typically requires special imaging conditions, for instance, the fluorophore blinking for STORM usually needs special hypoxic buffers and high illumination intensities, which may alter the specimen’s physiological state or binding of a fluorescent probe [61, 62]. There are already a handful of examples of using STORM for plant cell wall biology; some of the most notable are a recent study elucidating the role of pectin in pavement cell morphogenesis [16] and an application of multicolor 3D-STORM to study the assembly of three major cell wall components during nascent cell wall formation [63].

### Live cell imaging

Live cell imaging is important for studying biological processes, such as cell wall biosynthesis and remodeling, in real time [64, 65]. A widely used approach for visualizing the cell wall formation in living specimens combines the selective enzymatic removal of cell wall components with probe-assisted polysaccharide labeling. In one example, homogalacturonan (HG) secretion in the unicellular green algae *Penium* sp. was visualized in living cells via CLSM after treatment with pectate lyase and labeling with various antibodies. This showed that HG is secreted into certain cell wall areas as a highly methylated form before it becomes demethylated and cross-linked by $\hbox {Ca}^{2+}$ [66].

**Fig. 2:**
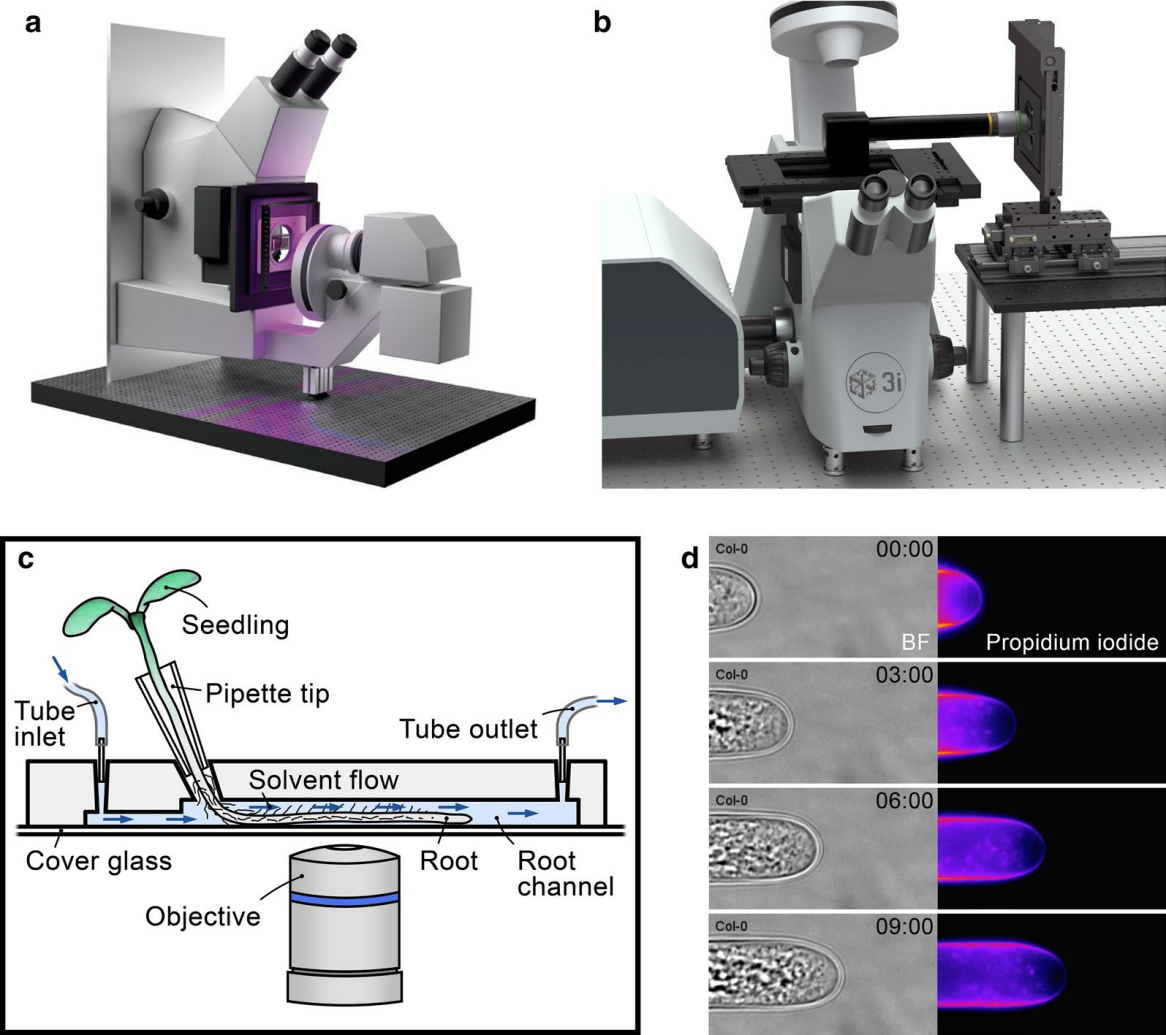
Strategies for imaging living plants in their natural, upright position or in a microfluidic device: **a** Inverted microscope mounted vertically on a metal plate, CC BY 4.0 [72]. **b** Periscope tube and a vertical sample stage [73] (Marianas Plant Scope with Spinning Disk, image courtesy of *3i Intelligent Imaging Innovations*). **d** Scheme of the RootChip [70]: Tubes connected to inlet and outlet allow for exposing the root to solvents during imaging. **d** Image series of an *Arabidopsis* root hair tip over 9 minutes, which is grown in the RootChip system. Pectin Ca^2+^ egg box complexes are visualized by staining with propidium iodide (right). Corresponding bright-field images are also shown (left) (Image adapted from Schoenaers et al. [71] with permission)

While unicellular organisms are relatively easy to image, visualizing cell wall dynamics in living land plants can be challenging. Sample mounting and protection usually requires special growth systems that allow for non-invasive imaging of intact plant organs such as roots. Optimally, such systems provide measures to control the environmental conditions during the experiment. Placing specimens in sealed chambered slides and/or under agar or phytagel is a straightforward measure to control humidity [67]. However, hypoxic responses during longer imaging sessions should be excluded, which can be, for example, tested for by using ADH:GFP plant lines [68]. An efficient strategy to establish controlled micro-environments is imaging plants in microfluidic devices [69]. For example, the RootChip allows for growing seedling roots in individual channels that can be infused with dyes and other liquids [70], see Fig. 2c. Advantageously, the RootChip can be directly mounted onto the microscope; thus the consequences of experimental treatments can be visualized immediately via time-lapse microscopy. Using this setup combined with propidium iodide staining (see "Small fluorescent molecular probes" section ) revealed that the cell wall formation in growing root hairs depends on an auxin-mediated oscillating demethylation of homogalacturonan (Fig. 2d; [71]). Major disadvantages of microfluidic devices are that their preparation can be laborious (e.g., setting up the RootChip takes $\sim$1.5 weeks) and that they are only compatible with small, young plants, isolated plant cells, or thallus cultures.

More advanced live cell imaging setups usually require special and/or customized microscopic equipment. As an illustration, plant roots are frequently used models to study various physiological or developmental processes. One way to monitor root biomass formation under a natural gravity vector is to use vertical imaging setups to counteract gravitropism. Customization usually starts from standard inverted CLSM systems, and in recent years, a number of laboratories published their imaging systems and described the customization steps in detail. This includes mounting a microscope on a metal plate, which can be flipped 90° [72] (see Fig. 2a), adding a periscope tube in the optical path [73] (see Fig. 2), or equipping a microscope with a special imaging chamber [46]. Flipping the whole microscope brings the advantage that all parts function as they do under normal orientation.

To study cell wall dynamics *in planta* with superresolution techniques, 2D or 3D SIM is typically chosen as the most compatible technique with the sample requirements of live cells. STORM and PALM are possible to use as well; however, longer imaging series might be limited due to photobleaching of fluorophores (for a recent review see Komis et al. [74]). Exciting new tools to overcome these limitations await introduction to the plant cell wall field; for example, the technique known as LIVE-PAINT was used to visualize cytoskeletal dynamics in living yeast below the diffraction limit and is compatible with standard confocal or TIRF microscopy systems [75]. It is based on the continuous imaging of genetically encoded, transiently binding small peptides, an approach that is expected to have high potential to track systems such as the dynamic microtubule-mediated cell wall biosynthetic machinery at high temporal and spatial resolutions.

### Autofluorescence in plants

In plant tissues, autofluorescence can be used for label-free cell wall imaging of phenolic-containing polymers like lignin or ferulates, but it can be obstructive when using exogenous fluorescent probes [76, 77], as their specific fluorescence has to be separated from the autofluorescence. This is typically done by intensity, where excitation and emission wavelengths are chosen so the dye is much brighter than the autofluorescence, but methods like time-gating [78] or fluorescence lifetime imaging [79] have also been used to distinguish fluorophores from the background autofluorescence. Additionally, spectral unmixing techniques can accurately correct for autofluorescence [80], and a variety of new linear, nonlinear, and unsupervised-learning algorithms have recently been developed that can make excellent use of multiwavelength imaging data to, for example, distinguish combinations of 16 different fluorophores in the same image [81], or unmix underdetermined signals without any reference input [82].

**Fig. 3:**
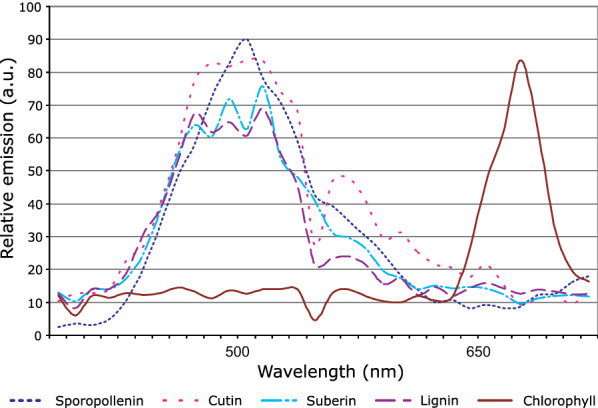
Emission spectra of autofluorescent molecules: fluorescence emission spectra of various components of *Arabidopsis* tissues, taken with two-photon excitation at 790 nM. Potential overlap with fluorophores is possible in the blue–green and red region of the visible spectra. Note that this spectra is very specific to the respective plant and tissue material. Adapted from Berg and Beachy [83] with permission

In photosynthetic cells, chlorophyll is the major source of autofluorescence, joined by the cell wall phenolics and other endogenous fluorophores such as ferulates, flavonoids, etc., recently extensively reviewed by Donaldson [84]. Figure 3 shows the emission spectra for many of these molecules in *Arabidopsis*, which falls mainly in the blue-green and red regions of the optical spectra. Therefore, fluorophores with green excitation laser and an emission peak in the yellow-red region should be preferred when imaging this plant tissue [85, 86]. It is important to note that the intensity of the autofluorescence depends very much on the imaging parameters (e.g., excitation wavelength, emission filters); therefore, it is recommended to image the plant tissue of an untreated wild-type plant with the imaging settings of the chosen fluorophores before staining plant material or transforming plants.

One way to simply remove autofluorescence from images is using the ClearSee or related protocols, designed to preserve fluorescent protein (FP) signals while optically clearing samples [87]. Although it is slow on thick samples, it is an inexpensive way to obtain excellent images of FP-based gene fusions and is compatible with several cell wall histological stains [88]. Some recent examples of its use are the simultaneous imaging of gene expression patterns and cell wall physiology of lateral root formation [89], or immune response [90] in *Arabidopsis* roots.

## Probes for fluorescence microscopy

### Small fluorescent molecular probes

Externally applied small fluorescent molecules or conjugates of fluorophores to other small molecules, like oligosaccharides or polyethylene glycol (PEG), can be used for direct labeling and/or real-time imaging of dynamics of cell wall polysaccharides and other non-carbohydrate cell wall components like lignin, suberin, and cutin [91, 92]. Many cell wall-specific fluorophores can be conveniently applied in conjunction with imaging of fluorescent proteins, enabling simultaneous visualization of FP-tagged proteins and cell wall components [88].

#### Examples in plants

**Fig. 4:**
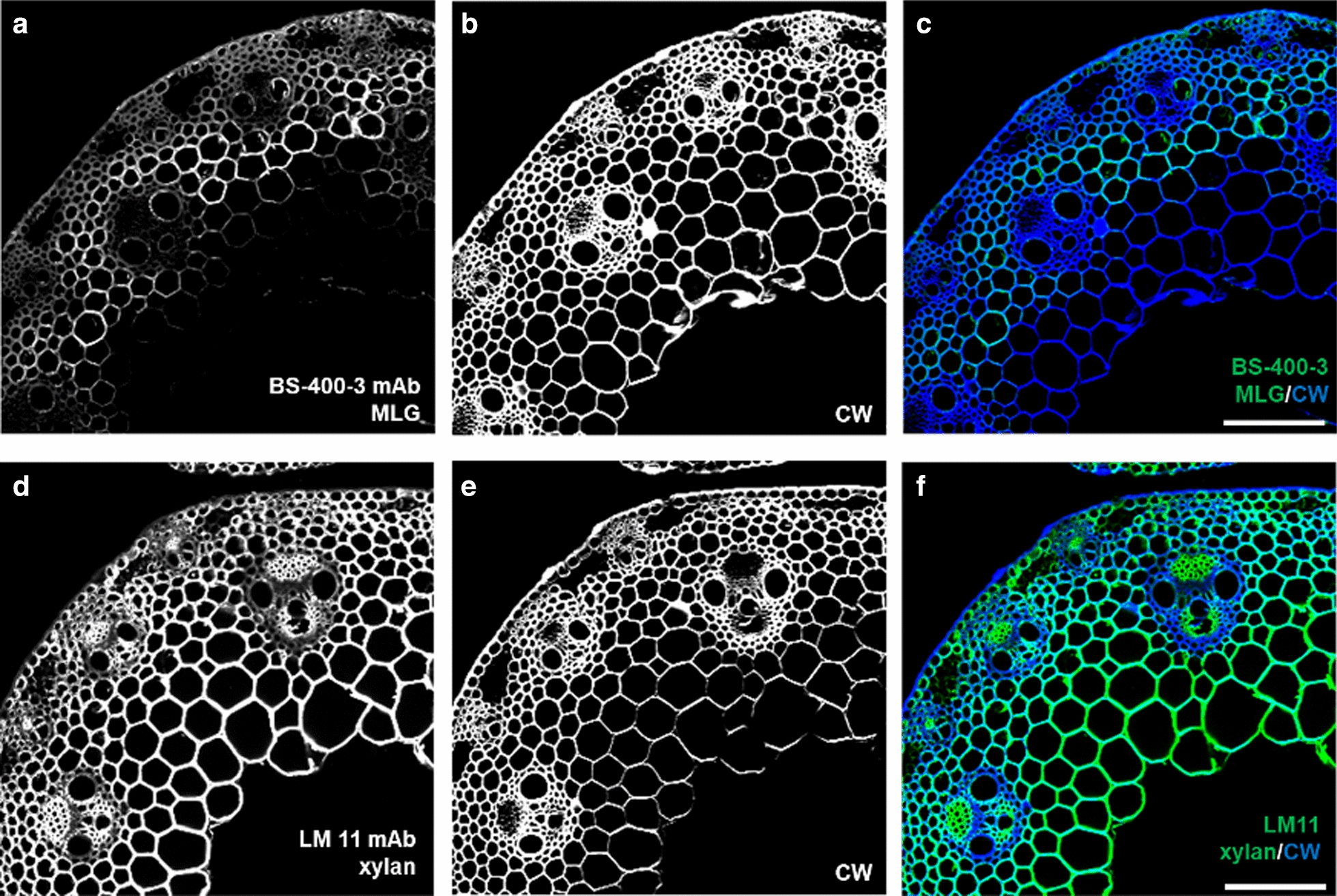
Examples of the application of anti-glycan mAbs for the characterization of cell walls. Labeling of cross-sections of a *Brachypodium* internode: **a**–**c** BS-400-3 antibody specific for mixed-linkage glucan (MLG; green signal). **d**–**f** LM11 specific for arabinoxylan (secondary anti-rat antibody conjugated to AlexaFluor488; green signal). **b**, **e** The sections were counterstained with Calcofluor White (CW; blue signal). Note the different distribution of the two epitopes. **c,f** Overlay of antibody and CW channels. Scale bars = 50 μm. Authors’ own unpublished work

The most used fluorescent dyes are traditionally those recognizing $\beta$-1,4-glucan. These include Calcofluor White (Fluorescent Brightener 28; Fig. 4) and Pontamine Fast Scarlet 4B (PS4B or Direct Red 23), the latter of which stands out as a more specific binder and possesses special properties including bifluorescence and compatibility with superresolution microscopy [42]. PS4B was used to study cellulose microfibril orientation in *Arabidopsis* epidermal cells [64], onion peel [42], and in tension wood [93]. In addition, many other dyes for cell wall polymers are available, for example, Solophenyl Flavine 7GFE, which exhibits specificity towards xyloglucan [64], basic fuchsin towards lignin, and Nile Red towards suberin [88].

There are also several reactive dyes that chemically react with either hydroxyl or aldehyde groups of a glycan moiety. Such an example is 5-(4,6-dichlorotriazinyl) aminofluorescein (5-DTAF), which reacts with hydroxyl groups at high pH. This dye was used to label cellulose microfibrils for superresolution microscopy [94] and to prepare fluorescent films for determination of fungal cellulase activity [95]. Superresolution-compatible imaging of cell wall carbohydrates oxidized with periodic acid was also recently demonstrated with the fluorophore ATTO 647N-amine, which reacts via a pseudo-Schiff reaction and was used to visualize cell wall ingrowths in phloem parenchyma transfer cells [96].

Fluorophores are also available for monitoring changes in physicochemical properties of the apoplastic moiety, such as 8-hydroxypyrene-1,3,6-trisulfonic acid (HPTS), which was used to study the pH response to auxin in roots [97]. The common dye propidium iodide (PI) can reversibly compete for binding with calcium ions that form complexes with negatively charged pectin chains, a property that has been used for live cell visualization of growth oscillations in root hairs and pollen tubes [98]. Additionally, new fluorescent molecules belonging to the class of luminescent conjugated oligothiophenes (LCO) have been found as suitable structure-sensitive probes for carbohydrate polymers, such as pentamer hydrogen thiophene ethyl amine (p-HTEA) and heptameric formic thiophene acetic acid (h-FTAA), which show a stereochemistry-dependent increase of fluorescence upon binding $\beta$-linked glucan targets [99, 100].

A new class of oligosaccharide-based fluorescent probes for pectin was introduced recently and exhibits stringent specificity for certain polyionic homogalacturonan (HG) regions. Specifically, fluorophore-functionalized chitosan oligosaccharides (COS) are able to bind to de-esterified HG by means of positive-negative charge pairing [101] and have also been applied to study HG distribution during lobe formation of the leaf epidermis [102]. Another type of oligosaccharide probes that bind targets in an ion-dependent manner are the recently developed pectin-recognizing long oligogalacturonides, which in the presence of calcium can form “egg box complexes” with endogenous, still-uncrosslinked HG chains [103]. This probe served well for fine structural analysis of the *Penium margaritaceum* intricate extracellular matrix [104].

Small-molecule dyes can also be incorporated into the cell wall polymers as substrates. For example, fluorescent monolignols can be polymerized into lignin macromolecules, allowing visualization of a cell wall polymer formation by radical coupling *in muro* [105]. This labeling strategy is the basis of a method known as bioorthogonal labeling imaging sequential strategy (BLISS), which uses a combination of different types of monolignols, fluorophores, and feeding times to study the progression of lignin formation over time [106].

Finally, although they are not usually considered along with small molecules, quantum dots (q-dots) are fluorescent semiconductor particles that are even brighter than most chemical dyes and are extremely photostable. Interestingly, (CdSe)ZnS q-dots naturally bind to hexahistidine affinity tags, which was used to study the distribution of carbohydrate-binding modules (CBMs) on a cellulose microfibril by identification of individual $\hbox {His}_6$-CBM-$\hbox {His}_6$ molecules [107].

#### Challenges

One of the most serious drawbacks of using small-molecule fluorescent probes is that their low molecular weight is usually associated with only limited specificity to glycan targets. In the case of reactive dyes, specificity is an even bigger issue as they usually target functional groups present in a large number of glycan moieties. In addition, some small-molecule fluorophores exhibit broad emission spectra and long Stokes shifts, complicating their use in multilabel experiments with the already limited window of wavelengths available for use in plants.

#### Future directions

Can we expect the introduction of new fluorescent probes in the future? Several fluorescent dyes were synthesized in the past as possible new dyes for natural materials in the textile industry, and these chemical libraries may be a valuable resource for novel probes with new specificities or applications, but many have not been characterized in detail. Other inspiration for new types of probes can be found in the animal biology field, where fluorescent glucose derivatives are commonly used as biosensors for tracking metabolic fluxes and glycogen biosynthesis [108]. Unfortunately, similar fluorescent analogs of the building blocks for cell wall polysaccharides are not yet available, but such molecules could be effective tools to study cell wall formation in living samples in a similar way as click chemistry-based metabolic labeling (discussed below in "Click chemistry labeling" section), without the necessity to perform the post-feeding fluorophore attachment.

### Anti-glycan antibodies

Anti-glycan monoclonal antibodies (mAbs) remain the most important molecular probes for *in situ* analysis of the plant cell wall structure ([92, 109, 110]; Fig. 4). Although the current set of antibodies is incomplete, it already covers a wide range of epitopes for the major classes of cell wall glycans and proteoglycans. The reader is directed toward previous reviews on anti-glycan antibodies [92, 109] and online databases [111, 112] for comprehensive lists of currently existing mAb specificities.

#### Examples in plants

Although research on detailed epitope characterization of antibodies using defined or synthetic glycan microarrays [113] now dominates over the generation of new antibodies, there are some notable recent additions to the toolbox of mAbs, for instance, a long-awaited monoclonal antibody against rhamnogalacturonan II (RG-II) [114].

#### Challenges

**Fig. 5:**
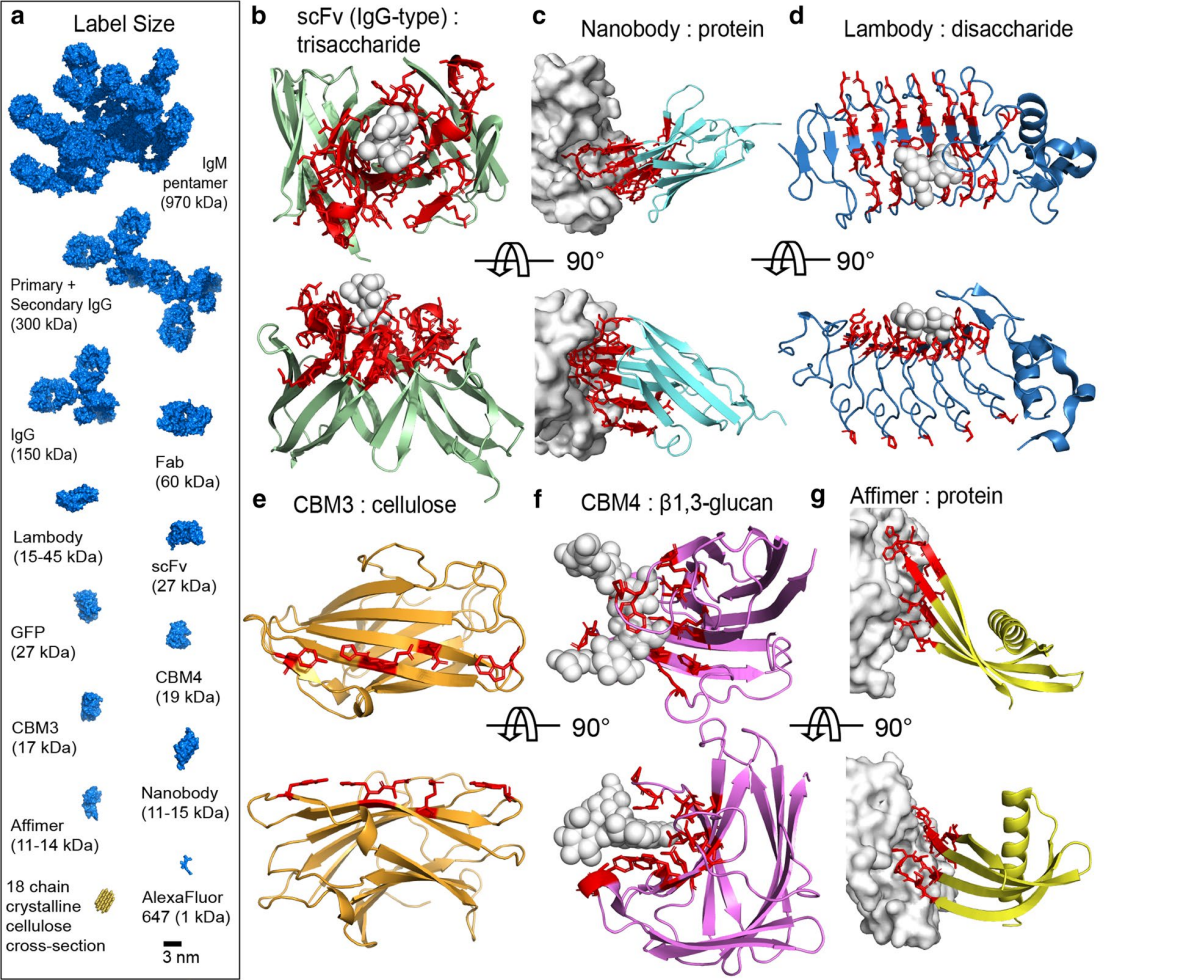
Comparison of proteinaceous labeling reagents. **a** Molecular size of labeling reagents. All figures drawn in PyMol from pdb accession codes 1IGY (IgG), 1BBD (Fab), 4K79 (Lambody), 1MFA (scFv), 1EMA (GFP), 1GUI (CBM4), 1NBC (CBM3), 3P0G (Nanobody), and 5MN2 (Affimer). The IgM structure is a hybrid model (IgM Fv and Cμ1-3 domains from EMD-4945 [135], and Cμ4 and J chain from pdb code 6KXS [136]) in a compact conformation bound to both antigen and complement proteins C1 and C4b (not displayed). Cellulose microfibril modeled with a 34443 habit [137]. **b** IgG-type domain of a scFv reagent with a trisaccharide antigen binding domain. CDR regions are shown in red sticks and the bound antigen in a white space-filling model. **c** Nanobody binding in a small cleft of a membrane receptor. CDR regions are shown in red sticks and the receptor in white surface model. **d** Lambody raised against a disaccharide antigen. Residues in positions with low sequence conservation are drawn in red sticks and the bound antigen in a white space-filling model. Note that this particular structure has 2 LRR-V repeats, but naturally occurring lambodies may have between 1 and 9 repeats. **e** CBM3a domain with the “planar strip” cellulose binding residues shown in red sticks. **f** CBM4 domain binding a $\beta$-1,3-glucan ligand. Ligand binding residues are highlighted as red sticks around the glucan in a white space-filling model. **g** Affimer binding to a protein ligand. Randomized residues are highlighted as red sticks with the protein target rendered in a white surface model

The size of the mAb is one of the limiting factors for the visualization of intricate features of the cell wall network, diagrammed in Fig. 5a. In particular, many plant glycan-binding antibodies are of the large, multivalent IgM subtype, which was raised as a potential source of discrepancies between a recent superresolution study of homogalacturonan rods via STORM [16] and data obtained by other methods, such as transmission electron microscopy (TEM) [17]. Epitope masking is also a common phenomenon observed for cell wall samples [110, 115, 116], a result of the tight association of the component polymers and/or the low porosity in some types of cell walls.

A major challenge with the development of novel anti-glycan antibodies is the limited immunogenicity of plant carbohydrates [92], and the potential cross-reactivity among structurally similar polymers. For instance, LM6 mAb recognizes arabinan structures on both rhamnogalacturonan I (RG-I) and arabinogalactan proteins (AGPs), requiring careful analysis and labeling with several different antibodies to ensure a correct identification of the signal origin [117].

#### Future directions

The generation of the first RG-II specific monoclonal antibody [114] and anti-starch mAbs [118] suggest some feasible approaches to overcome the limited immune response towards some carbohydrates. This includes using nontraditional animal hosts (see nanobodies below or lambodies in "Carbohydrate-binding modules and non-immunoglobulin scaffolds" section), synthetic antibody libraries, or “shotgun” immunization with complex, non-purified immunogens.

Inspiration for how to overcome the limitations due to the large size of the conventional immunoglobulins might come from biomedical research. Single-chain variable fragments (scFv) or single-domain antibodies (sdAb) are promising smaller alternatives that still use the same binding mode as traditional IgG and IgM reagents [119]. Additionally, a promising, relatively new antibody technology known as “nanobodies” can be used. These nanobodies are small (12 to 15 kDa), single-chain antibody fragments unique to camelids and cartilaginous fishes that contain their entire set of complementarity-determining regions (CDRs) on a single Ig-fold domain. Nanobodies create a more compact antigen recognition site which excels at binding to small, concave antigen surfaces that traditional antibodies, which typically bind with an interface between two separate sets of CDRs, often fail to recognize [120] (compare Fig. 5b and c).

Only a handful of examples of nanobodies against plant targets are available, such as those against *Arabidopsis* seed albumin and globulin [121], *Arachis hypogaea* glycinin (Peanunt allergen Ara h 3) [122], and *Ricinus communis* ricin toxin [123]. Additionally, several nanobodies that bind to *Chlamydomonas reinhardtii* cell walls at affinities of up to 1 nM were reported [124], although the epitopes they bind remains unknown. Encouragingly, this is a very active field, and the authors expect that nanobody generation and screening from synthetic phage-, yeast-, and ribosome-display libraries [125–127] will speed up their application to plant cell wall targets by removing the expensive llama/alpaca farms or shark aquariums from the development process.

### Carbohydrate-binding modules and non-immunoglobulin scaffolds

The substrate recognition moieties of carbohydrate acting enzymes (CAZYmes) are called carbohydrate-binding modules (CBMs) and are the second-most used proteinaceous probes for glycan targets. CBMs are particularly important for dealing with the lack of mAbs reactive towards cellulose. The characterization of the cellulose fraction is an essential step of biomass valorization because features such as crystalinity index (CrI) and degree of polymerization (DP) substantially affect saccharification efficiency [128]. CBMs may distinguish between crystalline and amorphous regions of cellulose [129, 130], however, binding specificity can still be a problem with CBMs. For example, CBM3a is one of the most popular probes for crystalline cellulose (see Fig. 5e), but it also recognizes xyloglucan [131]. A large set of CBMs ready to be conjugated to fluorescent molecules or purified as GFP fusions is now commercially available.

Besides CBMs, quite a few other types of non-antibody protein reagents are available for modification into new fluorescent probes. These scaffolds are chosen because of their favorable biochemical properties, such as thermal and pH stability, ease of expression, etc [132]. One type, known as affimers, is derived from a human or plant cystatins, a cysteine protease inhibitor. These $\approx$100 amino acid domains consist of a conserved $\alpha$-helix/$\beta$-sheet scaffold with two variable loops mediating target binding (see Fig. 5g) [119, 133]. While affimers present only a small variable domain and, like nanobodies, are not expected to be able to bind well to long, linear epitopes, they can be heat and pH stable, making them leading candidates for use in biotechnology applications that require robust reagents [134].

#### Examples in plants

CBMs have been widely applied to visualize the diversity of cellulose arrangements in plant tissues [138–140]. For example, CBMs are used for visualizing biomass deconstruction [28, 141] and characterization of cellulose alteration in cell wall mutants [142–144].

#### Challenges

Although CBMs have a diverse binding repertoire, a common drawback is that their specificity is often low [145]. This is particularly problematic when aiming to study the interaction of structurally similar polymers, for example, the association of cellulose and xyloglucan. Engineered protein reagents, such as affimers, can be made to specifically distinguish similar targets [146]; however, they have not yet been developed as polysaccharide probes.

#### Future directions

One popular method to find higher-affinity binding variants of protein domains like CBMs is phage display and directed evolution [147]. Traditional panning of random mutants with phage display has been used to modify the xyloglucan recognition activity of the CBM4 scaffold (see Fig. 5f) [148, 149], or the CBM11 scaffold [150]. However, using a directed evolution workflow of saturation mutagenesis on well-chosen sites of a CBM phage display library and performing analysis with next-generation sequencing (NGS) would allow determination of full residue substitution frequencies. A particularly instructive example of this process is that by Hu et al. [151], where NGS was used to find a 160x affinity enhancement of an existing antibody mutagenized in 5 CDR loops. The NGS data even allowed them to determine that one CDR converged back with the original sequence, an indication that this was already an optimal sequence for this antibody.

An interesting class of non-immunoglobulin scaffolds with well-established carbohydrate-binding activities are the variable lymphocyte receptors (VLRs) or “lambodies,” named after their function as antibodies in lampreys and hagfish. In a mechanism conceptually similar to that of mammalian B-cell receptors, the lamprey/hagfish lymphocyte-like cells assemble a lambody gene by selecting variable region domains randomly from a large set of possible sequences encoded by the germline, which code for turns of a leucine-rich repeat (LRR) domain [152]. The new randomized gene product folds into an elongated solenoid-type LRR protein, shown in Fig. 5d [153]. Note that similar LRR domains with a variety of lengths are prominent as pattern recognition receptors in both plant and animal innate immune systems, such as the binding domains of receptor-like kinases or toll-like receptors [154]. Lambody libraries have been proven to recognize animal glycosylation motifs with high affinity and specificity [155, 156], and due to their reactivity towards carbohydrates, lambodies have high potential to yield probes to challenging wall targets.

An underexplored method in understanding biomass utilization is imaging with directly-labeled CAZYmes or whole complexes of CAZYmes, such as cellulosomes. This approach has already been demonstrated with a few examples, such as with research on cell wall biomass deconstruction [28]. Besides cellulases, an inactivated xylanase was used to characterize the xylan distribution in the wheat endosperm [157]. We believe that this approach has unrealized potential; for instance, a completely new repertoire of hydrolytic enzymes specific for glycosidic bonds only found in RG-II have been identified and we believe these could be explored to generate specific RG-II probes [158].

### Click chemistry labeling

Click chemistry labeling is a useful tool for imaging of polysaccharides and other cell wall polymers *in situ* with a process that does not utilize genetic probes, but instead relies on small, metabolically-incorporated functional groups that are later conjugated to a suitable fluorophore. The click reactions that conjugate the fluorescent label are usually done at ambient temperature in an aqueous system with high specificity and reaction rates, and most can proceed without a catalyst. Furthermore, click reactions can be bioorthogonal, leading to low non-specific incorporation and allowing their use in live systems [159].

**Fig. 6:**
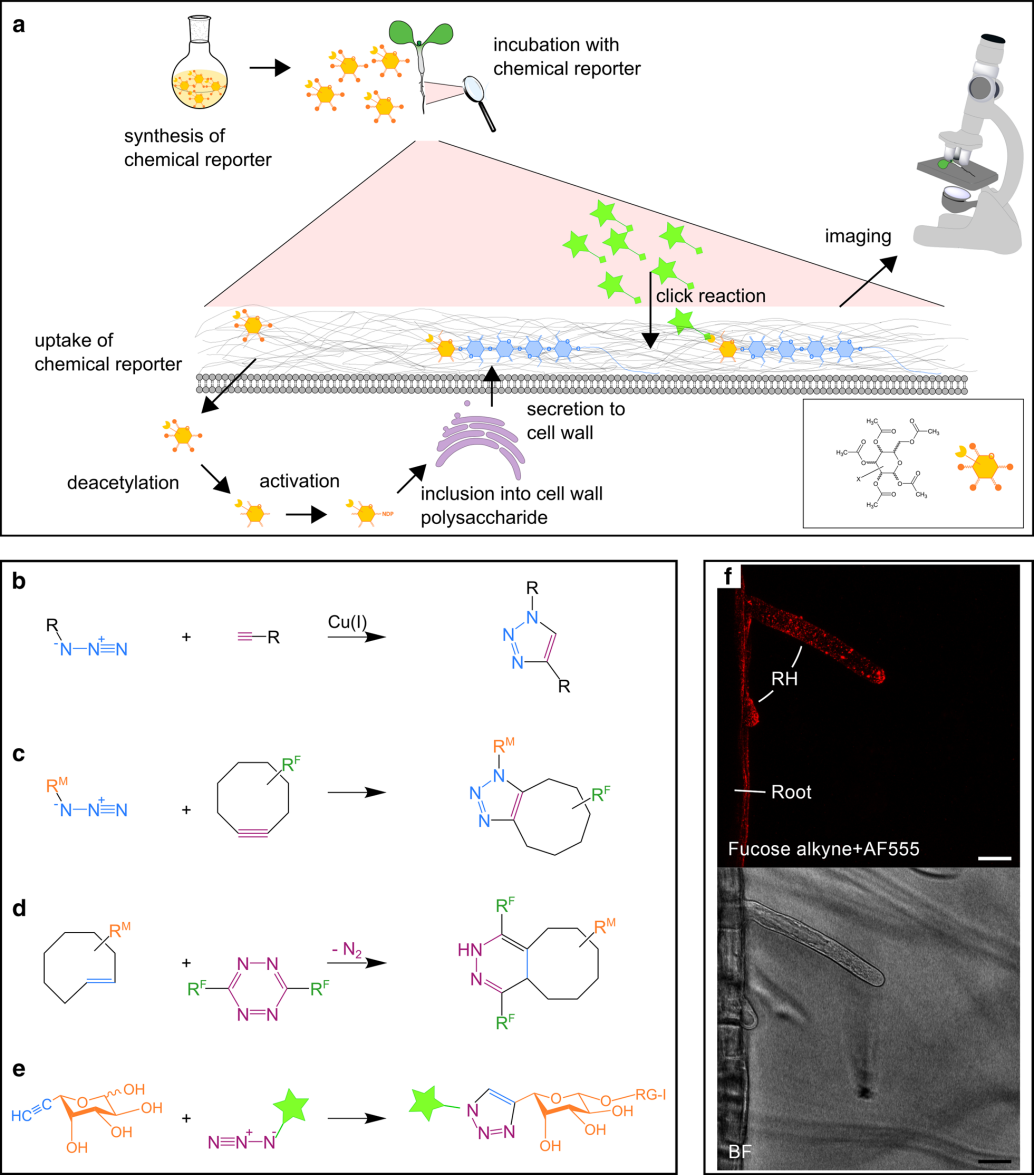
Scheme of the principle of click-labeling polysaccharides, common click reactions in plants, confocal image of a click-labeling experiment. **a** Scheme of the principle of click-labeling polysaccharides. The inset depicts an example of a functionalized and acetylated aldopyranose, the position of functionalization is not specified, and could be on of any of the hydroxy groups. $\text {R}^M$ monosaccharide, $\text {R}^F$ fluorophore, R either monosaccharide or fluorophore. **b**–**e** Common click reactions used in plant studies: **b** Cu(I)-catalyzed azide–alkyne cycloaddition (CuAAC), **c** strain-promoted alkyne-azide cycloaddition (SPAAC), **d** Inverse electron demand Diels-Alder ($\text {DA}_{inv}$). **e** Example of an incorporation and click reaction based on [164]. Note the small size of the triazole connecting the monosaccharide and the fluorophore. **f** Confocal (top) and corresponding bright-field image (BF, bottom) of fucose alkyne CuAAC click-labeled *Arabidopsis* root hairs (RH) and root, scale bar = 20 μm, authors’ own unpublished work, based on [164]

Classical click reactions, shown in Fig. 6b and e, proceed by cycloaddition of one azide- and one alkyne-containing substrate with each other, and either of these functional groups can be introduced as a modification to a monosaccharide of interest. Most of these modified sugars are commercially available as cell-permeable acetylated derivatives, which are taken up by the cell, deacetylated, and then activated and incorporated into a polysaccharide in the same manner as natural monosaccharides. A click-labeling reaction can be carried out with an opposite azide- or alkyne-functionalized fluorophore and imaged via optical microscopy, as depicted in Fig. 6a.

Three main reaction types are applied to plant cell wall research: (i) the classical Cu(I)-catalyzed azide–alkyne cycloaddition (CuAAC), (ii) the strain-promoted alkyne-azide cycloaddition (SPAAC), and (iii) the inverse electron demand Diels-Alder ($\hbox {DA}_{{inv}}$) reaction, see Fig. 6b–d and Table 1. The cytotoxicity of the Cu(I)-catalyst necessary for the CuAAC reaction can be problematic, which has driven the development and use of the latter two types of copperless reactions. The $\hbox {DA}_{{inv}}$ reaction is particularly fast and forms a strong, irreversible bond after $\hbox {N}_{{2}}$ release, but does require that a comparatively large moiety such as trans-cyclooctene (TCO) or 1-methylcyclopropenyl (1-MCP) are accepted as metabolic labels [160]. The tetrazine-containing reactive group is used as the fluorophore labeling reagent, as it is not stable enough under physiological conditions to be considered for cell feeding [161].

**Table 1:** The types of available click reactions between probe and reporter

Name	Chemical reporter	Probe	Bioorthogonal	Ref
CuAAC	Terminal alkyne	Azide	Copper toxicity	[162–164]
	Azide	Terminal alkyne	Copper chelating agent	[165]
SPAAC	Azide	Strained cycloalkyne	Yes	[166–168]
$\hbox {DA}_{{inv}}$	Strained/cyclic	Tetrazine	Yes	[168–170]
	Alkenes/alkynes			

*CuAAC* Cu(I)-catalyzed azide–alkyne cycloaddition,* SPAAC* strain-promoted alkyne-azide cycloaddition, ${DA}_{{inv}}$ inverse electron demand Diels-Alder

The advantage of click-labeling is that the bioorthogonal chemical reporter is a small functional group that often does not affect the functionalized monosaccharide’s ability to be incorporated into the cell wall polysaccharides. Many different monomers have been tested for their suitability for click-labeling in plants and are summarized in table 2. Although a large variety of metabolic labels and click-compatible fluorophores are commercially available, if needed, protocols for synthesis of new variants are reviewed in Sminia et al. [171].

#### Examples in plants

The pioneering work of click chemistry in plants was carried out by Anderson et al. in *Arabidopsis* roots [164], in which a fucose alkyne was likely incorporated into the pectin RG-I. Notably, this study could follow the dynamics of RG-I biosynthesis, allowing the authors to conclude that stronger fluorescence at the apical edges after 12 or 24 h is most likely due to reduced wall growth in that region. The same labeling strategy was used to investigation FRAGILE FIBER 1 (FRA1) in cell wall polysaccharides secretion [172] and is also depicted in Fig. 6f.

Incorporation of C7 azide-modified 3-deoxy-D-manno-oct-2-ulosonic acid (Kdo) into the pectin RG-II of *Arabidopsis* roots and tobacco BY-2 cells was studied by Dumont et al [173]. Furthermore, labeling of RG-I using alkyne-fucose and of cellulose with calcoflour white was optimized by the authors to examine colocalization of the RG-I, RG-II, and cellulose polymers. In another study, Hoogenboom et al. incorporated peracetylated, azido-modified *N*-acetylglucosamine, l-arabinose, l-fucose, and *N*-acetylgalactosamine ($\text{Ac}_4$GlcNAz, $\text{Ac}_3\text{ArabAz}$, $\text{Ac}_4\text{FucAz}$ and $\text{Ac}_4\text{GalNAz}$, respectively) into *Arabidopsis* roots [168]. Both CuAAC and SPAAC type labeling reactions were tested, revealing better labeling with the non-toxic SPAAC reaction.

Even though many alkyne and azido sugars were found to be incorporated without any obvious detrimental effect on growth, this is not always the case. For example, McClosky and coworkers investigated the incorporation of 6-deoxy-alkynyl glucose (6dAG) into *Arabidopsis* roots [174], where they found it arrested root hair growth and assimilated into emerging root hair tips and bulges. The authors suggest that 6dAG was incorporated into callose or another $\beta$-1,4 glucan, but the exact mechanism of the 6dAG toxicity remains unknown.

Several studies have also demonstrated click-labeling of lignins with modified monolignols [175–177], which are typically added to the incubation media of cut stems and are thus utilized as substrates during lignification. Multilabeling with different monolignols and monosaccharides was optimized by Simon et al. [178], who used a reaction sequence that ensured copperless reaction of azide groups before a final copper-mediated reaction of alkyne groups, a strategy that prevents crosslinking of adjacently incorporated azido- and alkynyl-monolingol substrates.

**Table 2:** Click chemistry-functionalized monosaccharides already applied to plants as chemical reporters

Monosaccharide	Click-reaction	Tissue	Compound	Live/fixed	Ref
Alkynyl fucose	CuAAC	Roots	RG-I	Cu(I) toxic	[164, 172]
Azido Kdo	CuAAC	Roots	RG-II	Cu(I) toxic	[173]
		Tobacco			
		BY-2 cells			
Alkynyl glucose	CuAAC	Root hairs	n.d.^a^	Arrests growth,	[174]
				Cu(I) toxic	
Azido Glc, Ara,	SPAAC	Roots	n.d.	Live	[168]
azido Fuc, Gal,	SPAAC			Live	
cyclopropene Glc	$\hbox {DA}_{{inv}}$			Live	
GlcNAz	CuAAC	Roots	N-linked	Cu(I) toxic	[179]
			Glycoproteins		
Alkynyl Fuc	CuAAC	Roots	n.d.	Cu(I) toxic	[180]
Azido Fuc, Kdo	SPAAC	Roots		Live	
GlcNAz	SPAAC	Roots		Live	
GalNAz	SPAAC	Roots		$\text {toxic}$^c^	
Alkynyl fucose	CuAAC	Flax stem	n.d.^b^	Cu(I) toxic	[178]

*n.d.* not determined,* Kdo* 3-deoxy-d-manno-oct-2-ulosonic acid.* Glc* glucose,* Ara* arabinose,* Fuc* fucose,* Gal* galactose.* GlcNAz* N-azidoacetylglucosamine,* GalNAz* N-azidoacetylgalactosamine

^a^ Incorporation hypothesized in callose or $\beta$-1,4 glucan by CSLD3.

^b^ Incorporation into non-cellulosic polysaccharides.

^c^ Moderate inhibition of root growth

#### Challenges

For monosaccharide labels, it is important to consider which position of the monosaccharide ring is modified, and if it supports correct linkage of the desired polymer [181]. Additionally, the metabolic label may inhibit internal transport processes or cause steric hindrance within the active sites of the specific enzymes that incorporate it into polysaccharides. However, these issues can be partially resolved by feeding of acetylated monosaccharides, which can diffuse across the plasma membrane and directly enter metabolite pools [174].

The type of click reaction used should be critically evaluated. For fixed samples, the small metabolic label size and established bioorthogonality of the CuAAC reaction is ideal, but in non-fixed samples, the CuAAC reaction damages the plant and may not keep the cell membrane intact during the labeling for some tissues [164, 172–174, 178–180]. It is clear that the SPAAC or $\hbox {DA}_{{inv}}$ reactions work better in this context, as they circumvent the necessity for a toxic copper catalyst, but there are many similar reagents for these reactions with little existing guidance on which is the best choice, so experimental optimization is still required for most live cell wall samples.

It is also important to determine which polysaccharides the chemical reporter gets incorporated into, as most monosaccharides are utilized for more than one polysaccharide [5, 182]. Moreover, epimerases or other metabolic enzymes may convert a functionalized monosaccharide into other products. For example, it is well established that in mammalian cells, *N*-azidoacetylgalactosamine (GalNAz) is epimerized into *N*-azidoacetylglucosamine (GlcNAz), and the same process seems to happen in plants that are unable to use GalNAz directly [168, 183]. To investigate the polysaccharide incorporation, extraction series and enzymatic treatments can be used to isolate the resulting labeled biopolymers. Biochemical characterization such as gel electrophoresis of carbohydrates (commonly known as PACE), or blots with SDS-PAGE for glycoprotein determination, can also be carried out [184, 185].

#### Future directions

Moving forward, it appears likely that more chemical reporters and click-labeling reactions will be developed or taken from animal cell research for use in plants. Recently, Wang et al. reported synthesis of glucose, mannose, rhamnose, and sucrose variants with new alkene and alkyne substitutions [186], and Zhu et al. published an extensive library of differently functionalized monosaccharides and aminosugars [180]. In the latter study, the authors observed successful labeling of *Arabidopsis* roots with differently functionalized versions of fucose, Kdo, *N*-acetylglucosamine, and *N*-acetylgalactosamine. Continuing work will be required to establish which of these and other currently available sugars and modification positions are tolerated by plants and can enter their metabolic networks.

We expect that current labeling strategies will be combined and optimized, for example, using two or three click reactions that are bioorthogonal and orthogonal to each other, such as those reported in Simon et al. [178]. Ideally, these reactions will not require washing steps between them, which takes additional time and may overly stress some tissues. Advancement towards such optimized multilabel chemistry is described by Tu et al., where tetrazine-nitrile and TCO-azide reactions specifically labeled their targets in a simplified, purified protein system [187], or in Wieczorek et al., where a range of tetrazine-fluorophores were employed for the $\hbox {DA}_{inv}$ without the need of washing steps [188]. We look forward to the application of such new reaction types and labeling reagents to plant samples, with many exciting options in development [189].

Click-labeling is not restricted to only biosynthesis and recycling of plant cell walls, but it can also be used to investigate enzyme and hormone activities. For example, the cellulosome of *Clostridium thermocellum* was characterized with a click-compatible substrate [190], or recently, cell wall auxin binding sites were discovered by means of click chemistry-compatible auxin analog [191]. Furthermore, progress is being made to track the site(s) of action of small molecules in cells using click chemistry probes [192], and similar approaches could be applied to study compounds that effect biomass processing, such as those having negative effects on saccharification processes or fermentation (e.g., enzyme inhibitors).

### Fluorescent protein tags

Fluorescent proteins (FPs) have become the most important fluorescent probes for live cell imaging (for extensive reviews of FPs see [193–195]) and have been successfully used for imaging in plants (see review [83]). Due to their relatively small size (22-28 kDa), they can often be fused to other proteins without interfering with their target’s cellular function.

Genetic engineering applications involving or modifying FPs are almost endless, many of which are compatible with fluorescent imaging. They have been turned into subcellular localization markers [196], biosensors [197], molecular rulers [198], colocalization markers [199], timers [200, 201], as well as essential components of PALM-type fluorescent superresolution imaging strategies [75]. To help scientists effectively utilize this large variety of FP variants, we direct the reader to an excellent open-source fluorescent protein database [202] that lists almost all available FPs and their properties; https://www.fpbase.org/.

#### Examples in plants

A prominent example using FP-tagged cell wall biosynthetic proteins was performed by Paredez et al. [203], where the association of the CesA complex (CSC) to cortical microtubules was visualized by fusing the citrine yellow FP to CesA6. In a subsequent study, the movement of the CSC along the microtubules could then be correlated to the catalytic activity of cellulose synthesis [204]. Imaging cell wall-resident proteins using FPs is fairly rare, but a recent example from Chou et al. using FP-tagged, lignin-related proteins allowed specific sections of the cell wall to be identified [205].

**Fig. 7:**
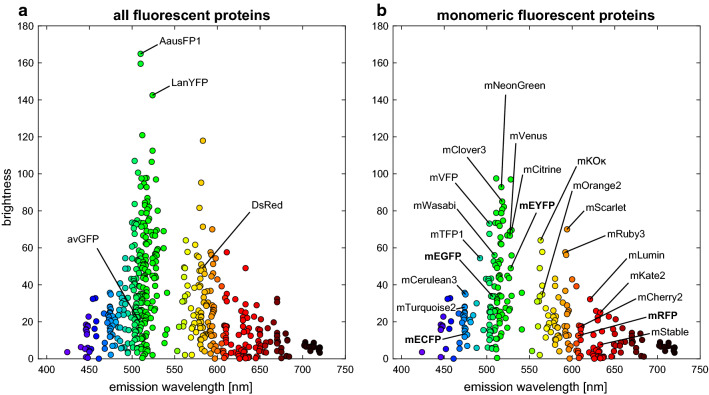
Overview of fluorescent proteins (FPs). **a** All FPs available from fpbase.org [202]. The original avGFP [206] and DsRed [207] FPs served as templates for most engineered FPs. LanYFP [208] was used to create mNeonGreen. AausFP1 [209] has recently be found to be the brightest wild-type fluorescent protein. **b** A brightness comparison of notable monomeric FPs

#### Challenges

Choosing the best FPs for imaging of cell wall-resident and biosynthetic proteins remains challenging, but it is well worth the effort to replace old FP technology with new variants that are better suited to the task. Newly discovered native fluorescent proteins (e.g., new FPs from *Aequorea* [209]) or enhanced FPs (e.g., a new photostable YFP variant [210]) are published almost every month (65 new FPs since 2018 [202]). Tools like FPbase [202] can help in finding the optimal FP for an application, but note that in general, FPs are either characterized *in vitro* [211, 212] or in mammalian systems [213, 214]. Additionally, the reader can find an extensive guide to choosing the right FP in Methods in Cell Biology [215].

There is no good reference for quantitative comparison of FPs in plants, partly due to the high biological variability in expression levels and silencing. An overview of the most commonly used FPs and other notable newcomers is shown in Figure 7. Note that most naturally occurring FPs form dimers (or even tetramers), which can drastically effect experiments that are sensitive to protein clustering. Figure 7b displays only monomeric FPs which have been engineered to avoid this oligomerization.

In the context of targeting FPs to the cell wall of plants, the following criteria should be considered:

- *Excitation/emission spectra* Due to strong autofluorescence in different plant tissues, FPs with cyan and far-red FPs emission wavelengths should be avoided (see "Autofluorescence in plants" section); note that pH can heavily influence both the FP and autofluorescence spectra and intensity [216]. For multicolor experiments without spectral unmixing, it is recommended to avoid a large overlap of the emission spectra, such as the mEGFP/mEYFP pair.
- *Brightness* FP brightness is usually calculated as the product of the extinction coefficient and the quantum yield, but it is important to note that these parameters are typically measured *in vitro* at the major excitation and emission peaks. The brightness of FPs in plant cells or subcelluar compartments can differ from published data [217]. Furthermore, without optimized excitation wavelengths and emission filters, even very bright FPs such as mNeonGreen might still appear darker than comparably dimmer FPs [213].
- *Photostability* All FPs undergo a photo-conversion process where they enter one or several dark states and are unable to fluorescence. This photobleaching behavior is hard to predict and can be inconsistent across different imaging methods (e.g., widefield vs. confocal detection), and unfortunately, the field lacks a reporting standard for photostability. If only single images are necessary, bright but fast-bleaching fluorophores like mScarlet can be used; if time-lapse images are required to observe dynamics, dimmer FPs like mCherry are more useful due to their slower photobleaching [214].
- *Sensitivity to pH* The pKa measurement describes the pH value at which fluorescence intensity drops to 50% of its maximum value due to chromophore protonation. Common FPs like mEGFP (pKa=6.0) and EYFP (pKa=6.9) will therefore loose most of their brightness when targeted to the cell wall [216]. On the other hand, FPs with low pKa values like mCherry (pKa=4.5) and TagRFP (pKa=3.8) are more stable in acidic environments and have been successfully imaged in the cell wall of plants [205, 218]. Several other FPs have been tested for cell wall-resident proteins in Stoddard et al. [216], where the authors recommend using mTurqouise2, mNeonGreen, or mCherry.
- *Non-fluorescent FP fraction* Not all FPs that are expressed in cells will be fluorescent. There is a considerable fraction of non-fluorescent proteins present (e.g., 20–40% for mEGFP [219]), which is mainly caused by slow maturation of the FP and long-lived dark states. The fluorescent fraction of FPs is not well characterized for many FP variants, but efforts are being made to characterize this parameter [214]. There are also “superfolder” versions of many FPs that will often help to prevent this problem [220, 221].

#### Future directions

While the highly active field of FP engineering for mammalian systems is geared towards brighter far-red dyes [195], this wavelength range is of more limited use for imaging in plants due to their autofluorescence. Instead, the focus for new FP development for cell wall imaging should be on making FPs more pH robust [222] and reducing their size [223]. Photo-activatable FPs like PA-GFP [224] and PA-mCherry [225] could also help to reduce background fluorescence in regions where proteins accumulate, as well as being an essential part of superresolution microscopy based on PALM [226] or the low-light techniques RESOLFT [227] and NL-SIM [228]. Currently, only a few photo-activatable and photo-switchable FPs are verified and optimized for use in plants [229], but more choices are needed.

FPs with large stokes shift like LSSmOrange and LSSmKate2 [230] show promise to help extend the useful emission wavelength window in plants by allowing blue-green light to be used to activate orange and red fluorescence. Bioluminescence imaging may also allow detection of blue or green while completely avoiding autofluorescence issues, such as the recent reports that use green enhanced nano-lantern and furimazine for whole-plant luminescence [231] and an approach that reconstitutes the fungal caffeic acid cycle to create an auto-bioluminescent plant [232].

### Aptamers

**Fig. 8:**
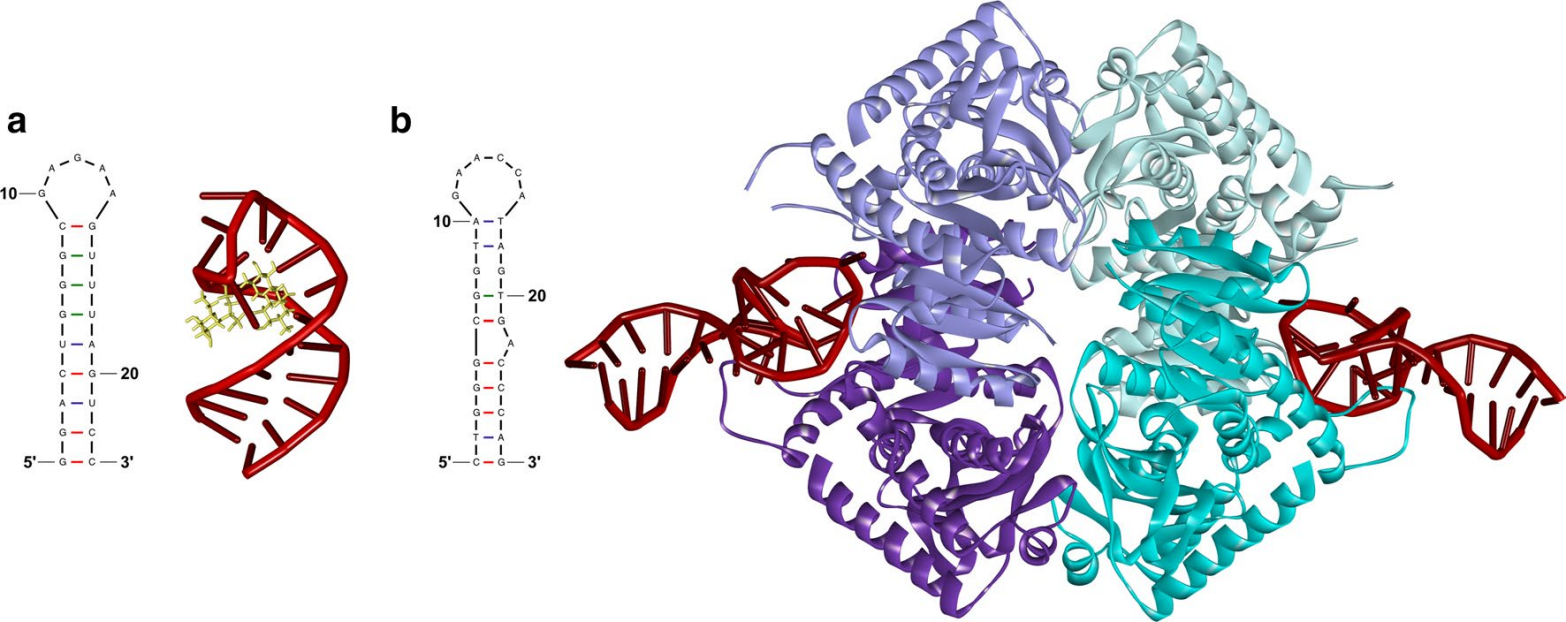
Aptamer structures and their interactions with the targets. **a** The 2D structure of RNA aptamer recognizing an aminoglycoside antibiotic, neomycin B, and the 3D structure of the aptamer (red) bound to the target (yellow) (PDB accession number: 1NEM). The stem of the RNA aptamer forms a pocket with the three consecutive $\text {G}\bullet \text {U}$ mismatches and parts of the loop interacting with the target. **b** The 2D model of 2008s ssDNA aptamer and 3D representation of two aptamers (red) bound to tetramer complex of *Plasmodium falciparum* lactate dehydrogenase (light and dark lilac and light and dark cyan) (PDB accession number: 3ZH2). The 2008s aptamer is able to recognize two distinct sites on the dehydrogenase molecule via interaction with different nucleotides. The 2D aptamer structures were generated using mfold web server and the 3D structures bound to the specific target were modified in Discovery Studio Visualizer

Nucleic acid-based probes, called aptamers, are another class of underutilized molecules to be explored for plant cell wall labeling. They are short oligonucleotides (ssDNA or RNA), usually 20-60 bp long, that form unique 3D structures which can bind their target with high affinity and specificity. Aptamer technology was introduced over 30 years ago [233, 234], and since then, it has gained popularity in pharmaceutical and environmental studies, enabling the detection of targets varying in size and chemical nature (see Fig. 8), all the way from ions to whole cells [235]. After an initial rush to use aptamers for medical technology went largely unrealized, their development continued in the field of biosenors, where aptamers towards small-molecule toxins and pathogenic bacteria have been found to be inexpensive testing reagents for water and food quality [236].

New aptamers are typically identified by *in vitro* evolution, a process known as Systematic Evolution of Ligands by Exponential Enrichment (SELEX). Various types of selection are available to assure identification of highly specific and selective aptamers, which is effective for challenging targets such as those with low immunogenicity [237]. The nucleic acid nature of aptamers allows for high screening densities, convenient library creation, and excellent chemical and thermal stability.

#### Examples in plants

Although several carbohydrate-binding aptamers with potential application in plant research have been developed, e.g., galactose, cellobiose, cellulose, and $\beta$-(1,3)-D-glucan among the targets, they have not yet been used in plant cell wall studies [238–240]. However, an RNA aptamer was recently used as an *in situ* gene expression reporter system in *N. benthamiana* and *Arabidopsis* [241].

#### Challenges

Unfortunately for cell wall applications, the heavy reliance on hydrogen bonding for standard nucleic acid interactions can lead to unsuccessful selection of aptamers that require binding with more hydrophobic or charged character. Because of this, much of the recent work towards improving aptamer selection is dedicated to increasing the chemical diversity of the component nitrogen bases [242]. One such modification that was successful for detection of blood glycoproteins is the modification of nucleotides with boronic acid [243], and boron chemistry has proven to be particularly useful for recognition of the adjacent hydroxyl functional groups on saccharide residues [244].

Careful consideration of application and optimization of the selection process are crucial for development of high affinity binders, comprehensive overview of the potential SELEX and post-SELEX pitfalls, and possible solutions to overcome them can be found in Wang el al. [245].

#### Future directions

A major benefit of using aptamer technology instead of protein-based labeling reagents is that their nucleic acid nature gives aptamers an unmatched versatility in downstream applications. For example, aptamers are modular, which allows them to be linked together for simultaneous detection of several targets, or addition of known fluorophore binding sequences can allow fast synthesis of fluorescent probes [246]. Modern molecular biology tools and inexpensive commercial synthesis of nucleic acid sequences can allow aptamers to be used directly as components in high-value biomass valorization, such as biosensor fabrication [247].

The combination of SELEX-based evolution and PCR-based amplification technology may allow microscopy to be used as an aptamer selection tool, such as with the “Morph-X-Select” technique used to evolve aptamers that associate with target cells isolated by laser dissection from native tissue sections [248]. Application of such a technique to plant tissue sections would mean any target that can be identified in a microscope (e.g., cell types, wall structures, specific cellular interfaces) could have labeling reagents made for it. Another interesting recent study reported the discovery of an aptamer that binds gluten while dissolved in the deep eutectic solvent ethaline [249]. Such solvents are promising candidates for efficient and environmentally friendly extraction of a variety of plant-derived compounds [250, 251], and development of aptamers that function in these solvents opens up new possibilities for understanding the dissolution process on detailed, molecular level.

## Conclusion

In this review, we discussed both well-established and state-of-the-art techniques for fluorescent probing and imaging of plant cell walls and plant biomass. We attempted to highlight possible future directions in this dynamic research field. Fluorescence microscopy analysis has been pivotal for our understanding of mutants in biosynthetic genes, the characterization of plant varieties, and for monitoring the stages of biomass deconstruction. However, there are still many gaps in our understanding of plant cell wall diversity, how different cell wall components are synthesized, how these components are then assembled into the cell wall, and finally how these elaborate structures are dynamically modulated during the life of a plant. Filling these gaps would be not only beneficial for understanding plant biology but also for setting directions in bioengineering and designing biomass processing for its efficient conversion into biofuels and high-value chemicals.

Many probing tools are focused on selectively detecting the structures built from cell wall components, but very few technologies are able to detect the numerous bonds between the various cell wall components. These bonds are of both non-covalent (ionic interactions, hydrogen bonds, Van der Waals forces) and covalent nature and are the ‘rivets’ holding the cell wall structure together. In many instances, these interactions are the key factors determining the level of biomass recalcitrance. NMR techniques have enabled an appreciation of the biochemical and structural nature of interaction of some of the polymers, especially of xylan, lignin, and cellulose. However, the possibilities to study and detect these interactions *in situ* are still limited, and development of fluorescent probes recognizing such interlinks would greatly help our understanding of how the individual polymer components of cells wall are ultimately assembled into whole, functional tissues and organisms. The problem is not straightforward and likely requires non-conventional approaches, such as *in vitro* evolution and selection of probes.

Although the current immunological toolbox for creating molecular fluorescent probes is relatively extensive, some classes of specificities are largely missing, such as probes to discriminate the level and patterns of acetylation in the cell wall. Additionally, the non-glycan cell wall components are generally underrepresented, such as waxes and suberin. Last but not least, algae are also an emerging potential source of biomass production with a high abundance of polysaccharides, especially of sulfated polymers. However, they are very biochemically diverse and the current repertoire of molecular probes for these less well-studied polysaccharides is still relatively small.

Cell wall-resident proteins like expansins, xyloglucan transglycosylases/hydrolases (XTHs), or pectin methylesterases (PMEs) are instrumental for assembly and spatiotemporal cell wall control. However, surprisingly little is known about their precise localization or movement within the cell wall. Probes against some of these cell wall proteins will be instrumental to understand the link between the cell wall protein activity, localization and formation of the cell wall microdomains, and whether modulation of these proteins can be used to design cell walls with desired properties. Fortunately, genetic fusion of fluorescent proteins with these enzymes and proteins is also possible, allowing a powerful, alternate approach to fluorescent imaging for these relatively low-abundance but very important cell wall components.

When it comes to imaging technologies, we are certain that superresolution microscopy will be used more in the coming years, in conjunction with new and better fluorescent probes. It is then expected that new features of the cell wall organization will be resolved that were previously out of reach for conventional widefield or confocal microscopy. Additionally, we hope that more effort will be made toward the underexplored combinations of fluorescent microscopy and other visualization techniques such as electron microscopy or atomic force microscopy. Currently, cell walls are typically imaged as 2D scans of a sectioned material, but we anticipate that progress in implementing tomographic methods and 3D fluorescent imaging will give us new information about the spatial organization of plant tissues and the cell wall in 3D, or even the 4D organization through time in live cells.

In conclusion, although the field of plant biotechnology is often lagging behind the biomedical field when it comes to imaging technologies, we expect that future implementation of new, exciting, and innovative imaging approaches will bring better understanding of the biology of plant cell walls and will serve the effective utilization of one of the most important renewable energy resource—cell wall-derived biomass.

## Data Availability

Reagents and data for original research reported here and files for figure creation are available upon reasonable request to the authors.
